# 伴NFE2基因突变骨髓增殖性肿瘤患者的临床和分子生物学特征分析

**DOI:** 10.3760/cma.j.cn121090-20241214-00569

**Published:** 2025-10

**Authors:** 颂扬 赵, 冰 李, 泽锋 徐, 铁军 秦, 士强 曲, 丽娟 潘, 蒙 焦, 清妍 高, 慧君 王, 琦 孙, 玉娇 贾, 怡汝 闫, 婧晔 龚, 富慧 李, 昕 王, 志坚 肖

**Affiliations:** 1 中国医学科学院血液病医院（中国医学科学院血液学研究所），血液与健康全国重点实验室，国家血液系统疾病临床医学研究中心，细胞生态海河实验室，天津 300020 State Key Laboratory of Experimental Hematology, National Clinical Research Center for Blood Diseases, Haihe Laboratory of Cell Ecosystem, Institute of Hematology & Blood Diseases Hospital, Chinese Academy of Medical Sciences & Peking Union Medical College, Tianjin 300020, China; 2 天津医学健康研究院，天津 301600 Tianjin Institutes of Health Science, Tianjin 301600, China

**Keywords:** 骨髓增殖性肿瘤, NFE2基因, 突变, 临床特征, Myeloproliferative neoplasms, NFE2 gene, Mutation, Clinical features

## Abstract

**目的:**

探讨伴NFE2基因突变的骨髓增殖性肿瘤（MPNs）患者的临床和分子生物学特征。

**方法:**

回顾性分析2021年4月至2023年6月期间就诊于中国医学科学院血液病医院接受血液肿瘤相关基因靶向二代基因测序的723例MPNs患者临床和实验室资料，分析伴NFE2基因突变患者的临床和分子生物学特征。

**结果:**

723例MPNs患者中，41例（5.7％）患者检出NFE2基因突变，NFE2基因突变中移码突变最常见（44.4％），其次是无义突变（33.3％）。NFE2基因突变组患者中位基因突变个数多于无NFE2基因突变组［4（2，5）个对2（1，3）个，*P*<0.001］。NFE2基因突变常与MPL、ATM、PPM1D、TET1基因突变共同发生。NFE2基因突变多为亚克隆突变，80.5％在MPNs驱动突变（JAK2、CALR、MPL）后发生。与无NFE2基因突变组比较，NFE2基因突变组年龄大［中位年龄：60（54，67）岁对54（41，63）岁，*P*＝0.001］，诊断前血栓病史（39.0％对22.0％，*P*＝0.012）、诊断前动脉血栓病史患者比例（36.6％对20.4％，*P*＝0.014）高。利用Logistic回归分析模型，校正年龄、合并症（慢性感染、肿瘤、自身免疫性疾病）后结果显示NFE2基因突变是TNF-α（*OR*＝2.747，95％ *CI*：1.143～6.605，*P*＝0.024）、γ干扰素（IFN-γ）（*OR*＝2.689，95％*CI*：1.191～6.076，*P*＝0.017）、IL-10（*OR*＝3.219，95％*CI*：1.343～7.717, *P*＝0.009）、IL-12P70（*OR*＝3.397，95％*CI*：1.003～11.508，*P*＝0.049）、IL-17（*OR*＝2.284，95％*CI*：1.017～5.127，*P*＝0.045）增高的独立影响因素。NFE2基因突变的真性红细胞增多症（PV）患者中国际工作组PV预后积分系统（IWG-PV）和包含基因突变的PV预后积分系统（MIPSS-PV）高危患者比例高（IWG-PV：66.7％对25.3％，*P*＝0.033；MIPSS-PV：22.2％对2.0％，*P*＝0.013）；原发性血小板增多症（ET）患者中MIPSS-ET高危患者比例高（15.4％对6.1％，*P*＝0.021）。NFE2基因突变（38例）和无NFE2基因突变组（671例）MPNs患者总生存期和累计血栓发生率差异均无统计学意义（均*P*>0.05）。

**结论:**

MPNs中NFE2基因突变多为移码突变。NFE2基因突变与MPNs患者年龄较大、多个炎症因子（TNF-α、IFN-γ、IL-10、IL-12P70、IL-17）水平升高相关，且多为MPNs疾病晚期出现的分子生物学异常改变。

## Abstract (en)

**Objective:**

To explore the clinical features and molecular characteristics of myeloproliferative neoplasms（MPNs）patients with NFE2 gene mutations.

**Methods:**

Gene targeted sequencing was used to detect NFE2 gene mutation in 723 patients diagnosed with MPNs who were admitted to Institute of Hematology & Blood Diseases Hospital, Chinese Academy of Medical Sciences & Peking Union Medical College between April 2021 and June 2023. The association between NFE2 gene mutations and clinical features and molecular characteristics of MPNs patients were retrospectively analyzed.

**Results:**

Among 723 patients with MPNs, NFE2 gene mutations were found in 41 cases（5.7％）. NFE2 gene mutations were predominantly frameshift mutations（44.4％）, followed by nonsense mutations（33.3％）. The median number of mutations in patients with NFE2 gene mutations（4 [2,5]）was higher compared to the group without NFE2 gene mutations（2, [1,3]）（*P*<0.001）. NFE2 gene mutations frequently co-occurred with mutations in MPL, ATM, PPM1D, and TET1. NFE2 gene mutations were mostly sub-clonal events, with 80.5％ occurring after MPNs driver mutations（JAK2, CALR, or MPL）. NFE2 mutations were correlated with older age［median age: 60（54, 67）years *vs* 54（41, 63）years, *P*＝0.001］. Patients with NFE2 gene mutations had a higher incidence of pre-diagnosis thrombosis（39.0％ *vs* 22.0％, *P*＝0.012）and pre-diagnosis arterial thrombosis（36.6％ *vs* 20.4％, *P*＝0.014）. Using a logistic regression analysis model adjusting for age and comorbidities（including chronic infections, malignancies, and autoimmune diseases）, NFE2 gene mutation was identified as an independent determinant of elevated tumor necrosis factor-alpha（TNF-α）（*OR*＝2.747, 95％*CI*:1.143–6.605, *P*＝0.024）, interferon-gamma（IFN-γ）（*OR*＝2.689, 95％*CI*:1.191–6.076, *P*＝0.017）, IL-10（*OR*＝3.219, 95％ *CI*:1.343–7.717, *P*＝0.009）, IL-12P70（*OR*＝3.397, 95％*CI*:1.003–11.508, *P*＝0.049）, IL-17（*OR*＝2.284, 95％*CI*:1.017–5.127, *P*＝0.045）. In polycythaemia vera（PV）patients with the NFE2 gene mutation, the proportion of those classified as high-risk is notably higher in both the IWG-PV and mutation-enhanced international prognostic systems for PV（MIPSS-PV）（66.7％ *vs* 25.3％ for IWG-PV, *P*＝0.033; 22.2％ *vs* 2.0％ for MIPSS-PV, *P*＝0.013）. Similarly, for essential thrombocythaemia（ET）patients, the proportion in the high-risk group of the mutation-enhanced international prognostic systems for ET（MIPSS-ET）is significantly higher（15.4％ *vs* 6.1％, *P*＝0.021）. No statistically significant differences were observed in overall survival or cumulative incidence of thrombosis between NFE2-mutated（38 cases）and non-mutated MPNs patients（671 cases, *P*>0.05）.

**Conclusion:**

NFE2 gene mutations in MPNs were predominantly frameshift mutations. NFE2 gene mutations were correlated with older age, elevated levels of several inflammatory factors（including TNF-α、IFN-γ、IL-10、IL-12P70、IL-17）, and they mostly occurred in late-stage of MPNs.

骨髓增殖性肿瘤（MPNs）是一组起源于造血干细胞的髓系肿瘤性疾病，近年非驱动基因突变对MPNs预后影响的研究报道越来越多[1]–[5]。有文献报道[6]，非驱动基因NFE2突变的小鼠表现为MPNs表型并可向急性白血病和（或）髓系肉瘤转化。2％～5％的MPNs患者伴有NFE2基因突变[7]–[10]，关于伴NFE2基因突变MPNs患者的临床和实验室特征，以及NFE2基因突变对患者血栓发生、生存等影响的研究较少。本文回顾性分析723例MPNs患者NFE2基因突变频率、突变特征及对患者临床结局的影响，现报道如下。

## 病例与方法

1. 病例：纳入本研究的病例为2021年4月到2023年6月在中国医学科学院血液病医院诊断的Ph染色体阴性的MPNs患者。纳入标准：①符合世界卫生组织（WHO）第五版诊断分型标准[11]；②接受包含267个血液肿瘤相关基因靶向二代测序。共723例患者纳入本研究，其中真性红细胞增多症（PV）159例（22.0％）、原发性血小板增多症（ET）260例（36.0％）、原发性骨髓纤维化（PMF）234例（32.4％）、PV/ET后骨髓纤维化（post-PV/ET MF）70例（9.7％）。男性324例（44.8％），女性399例（55.2％）。本研究经中国医学科学院血液病医院伦理委员会批准（批件号：KT2020035-EC-2），所有患者均知情同意，本研究符合《赫尔辛基宣言》的要求。

2. 细胞因子检测：留取患者外周血，分离血浆，应用江西省赛基生物技术有限公司细胞因子联合检测试剂盒（免疫荧光法），在贝克曼库尔特Navios流式细胞仪检测12种细胞因子［白细胞介素（IL）-1β、IL-2、IL-4、IL-5、IL-6、IL-8、IL-10、IL-12P70、IL-17、TNF-α、干扰素（IFN）-α、IFN-γ］。实验过程严格按照试剂盒说明书进行。

3. 染色体核型分析：骨髓细胞24 h培养后收集细胞常规制片，G显带，染色体核型描述按照《人类细胞遗传学国际命名体制（ISCN2013）》。

4. 二代测序和突变时序分析：留取患者的外周血或骨髓样本，分离出单个核细胞并提取DNA。使用PCR引物扩增目的基因组（包含267个血液系统肿瘤相关基因），对目的区域DNA富集，采用Illumina测序平台测序。测序后的原始数据利用CCDS、人类基因组数据库（HG38）、dsSNP（v138）、1000genomes、COSMIC、PolyPhen-2等数据库进行生物信息学分析，筛选出致病性基因突变位点。

在携带≥2个基因突变的患者中，等位基因突变频率（VAF）最大的基因突变定义为主克隆突变。若其他突变与其VAF差值<5％，定义为共同主克隆突变；若VAF低于主克隆突变≥5％，则定义为亚克隆突变[12]。利用VAF判断其他突变与NFE2突变的相对时序，对于仅有1个NFE2基因突变的患者，以NFE2基因突变VAF为参照，其他基因突变高于其VAF>5％判断为早于NFE2基因突变，差值<5％判为同时突变，低于NFE2基因突变VAF≥5％则其晚于NFE2基因突变；若携带2个NFE2基因突变，则以VAF较高者为首次NFE2基因突变，较低者为第2次NFE2基因突变，共突变VAF高于首次NFE2基因突变≥5％，判为早于NFE2基因突变；与首次或第2次NFE2基因突变的VAF差值<5％，定义为同时突变；低于第2次NFE2基因突变，VAF差值≥5％，判其为晚于NFE2基因突变[13]–[14]。本研究中，截短突变定义为导致编码蛋白产物长度缩短的突变类型，包括无义突变、移码突变。

5. 预后评分系统：PV患者按照国际工作组PV预后积分系统（IWG-PV）[15]和包含基因突变的PV预后积分系统（MIPSS-PV）[4]分别进行预后评估。ET患者依次按照ET国际预后积分系统（IPSET）[16]和包含基因突变的ET预后积分系统（MIPSS-ET）[4]分组。PMF患者依据动态国际预后积分系统（DIPSS）[17]、针对中国PMF特征修订的DIPSS积分（DIPSS-Chinese）[18]、DIPSS-plus[19]、年龄≤70岁人群的基于突变改进的国际预后积分系统（MIPSS-70）[20]、MIPSS70+version2[5]、遗传学预后积分系统（GIPSS）[21]判断预后。此外，post-PV/ET MF患者按照PV和ET后骨髓纤维化预后模型（MYSEC-PM）[22]进行预后评分。

6. 随访：末次随访截止时间为2024年10月24日，随访信息来自门急诊病例、住院病历或电话随访。对随访期间死亡病例，根据病例或与患者家属电话联系确认。累计血栓发生率定义为从疾病诊断日期至首次发生血栓、末次随访或死亡日期为止，患者累积发生血栓的概率。总生存（OS）期定义为疾病诊断至死亡或末次随访时间。

7. 统计学处理：统计分析采用SPSS 27.0统计软件，绘图使用Graphpad prism 10.1.2软件。非正态分布的连续性变量用中位数和四分位数间距（*Q*_1_，*Q*_3_）描述，分类变量采用例（％）描述。利用Mann-Whitney *U*检验比较非正态分布的连续性变量差异，采用卡方检验或Fisher确切概率法比较分类变量差异。利用多因素Logistic回归模型，校正年龄和合并疾病后评估NFE2基因突变与细胞因子水平的关系。Kaplan-Meier法绘制累计血栓发生率与OS曲线，并利用Log-rank检验进行组间比较。双侧检验*P*<0.05为差异具有统计学意义。

## 结果

1. NFE2基因突变频率及位点分布：本研究共纳入723例MPNs患者，41例（5.7％）患者检出45个NFE2基因突变（图1），其中4例患者伴2个NFE2基因突变。PV、ET、PMF和post-PV/ET MF患者NFE2基因突变频率分别为5.7％（9/159）、5.0％（13/260）、6.0％（14/234）、7.1％（5/70），各诊断亚组间NFE2基因突变频率差异无统计学意义（*P*＝0.873）。

19例（46.3％）患者NFE2基因突变位于N端转录激活域，5例（12.2％）患者突变位于DNA结合域，10例（24.4％）患者突变位于两者之间。45个NFE2基因突变中，多数为移码突变（20个，44.4％），其次是无义突变（15个，33.3％），错义突变（6个，13.3％）。本研究突变最常发生于第261号密码子，即E261Afs*3突变（5个）。此外，本研究中第65号密码子的突变亦常见（Y65* 4个，Y65Lfs*20 1个）。

2. NFE2基因共突变情况和突变时序分析：NFE2基因突变和无NFE2基因突变组中位突变数量分别为4（2，5）个、2（1，3）个（*P*<0.001），两组携带中位非驱动突变数量分别是3（1，4）个、1（0，2）个（*P*<0.001）。

全部723例MPNs患者中突变比例≥4％基因突变有JAK2（69.2％）、ASXL1（18.3％）、CALR（16.2％）、TET2（14.0％）、DNMT3A（9.8％）、NFE2（5.7％）、MPL（4.3％）、SF3B1（4.0％）。NFE2基因突变的患者频率较高的基因突变依次为：JAK2（63.4％）、CALR（24.4％）、DNMT3A（19.5％）、TET2（14.6％）、MPL（12.2％）、ASXL1（9.8％）、ATM（9.8％）、SF3B1（7.3％）、TP53（7.3％）、PPM1D（7.3％）、NF1（4.9％）、KMT2C（4.9％）、KMT2D（4.9％）、PDS5B（4.9％）、TET1（4.9％）。与无NFE2基因突变患者相比，NFE2基因突变患者更可能合并MPL（12.2％对3.8％，*P*＝0.026）、ATM（9.8％对2.1％，*P*＝0.015）、PPM1D（7.3％对1.8％，*P*＝0.048）、TET1（4.9％对0.1％，*P*＝0.009）基因突变；ASXL1、SRSF2、IDH1/2、U2AF1、EZH2、TP53、SF3B1、NRAS、KRAS、CBL、RUNX1等MPNs预后不良基因的突变频率在两组间差异无统计学意义（均*P*>0.05）；驱动基因JAK2V617F、JAK2exon12，CALR、MPL中位VAF在两组间差异也无统计学意义（均*P*>0.05）。

**图1:**
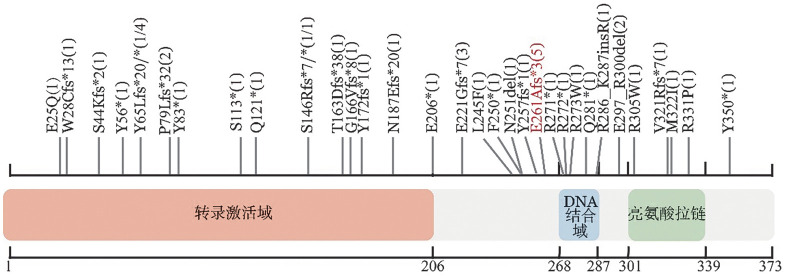
41例骨髓增殖性肿瘤（MPNs）患者NFE2基因突变氨基酸位点分布 **注** 横坐标表示NFE2蛋白氨基酸序列位置，不同颜色区域对应功能结构域：转录激活域（红色），DNA结合域（蓝色）和亮氨酸拉链结构域（绿色）；图中标注为41例MPNs患者检测到的NFE2突变位点，括号内数字表示突变患者例数，标红部分表示本研究中出现频率最高的位点

患者总体NFE2基因突变中位VAF为8.7％（4.0％，23.2％），PV患者NFE2基因突变中位VAF为7.8％（1.8％，19.4％），ET患者NFE2基因突变中位VAF为5.0％（3.0％，15.7％），PMF和post-PV/ET MF的中位VAF分别为12.9％（6.0％，36.1％）、19.9％（5.7％，32.7％）。各诊断亚组间NFE2基因突变VAF差异无统计学意义（*P*＝0.241）。

41例NFE2基因突变患者均同时伴其他基因突变，45个NFE2基因突变中37个（82.2％）是亚克隆突变，提示NFE2基因突变在MPNs中多为较晚发生的分子生物学事件。进一步分析NFE2基因突变与共同基因突变相对时序（图2），与NFE2基因突变伴随的117个突变中，56.4％（66个）早于NFE2基因突变发生，30.8％（36个）与NFE2基因突变同时发生，12.8％（15个）晚于NFE2基因突变。早于NFE2基因突变的基因主要是驱动突变（JAK2、CALR、MPL），常与NFE2基因突变同时发生的突变依次是ASXL1、DNMT3A、TET2。NFE2和驱动基因的相对时序分析，80.5％ MPNs驱动突变早于NFE2基因突变。

**图2:**
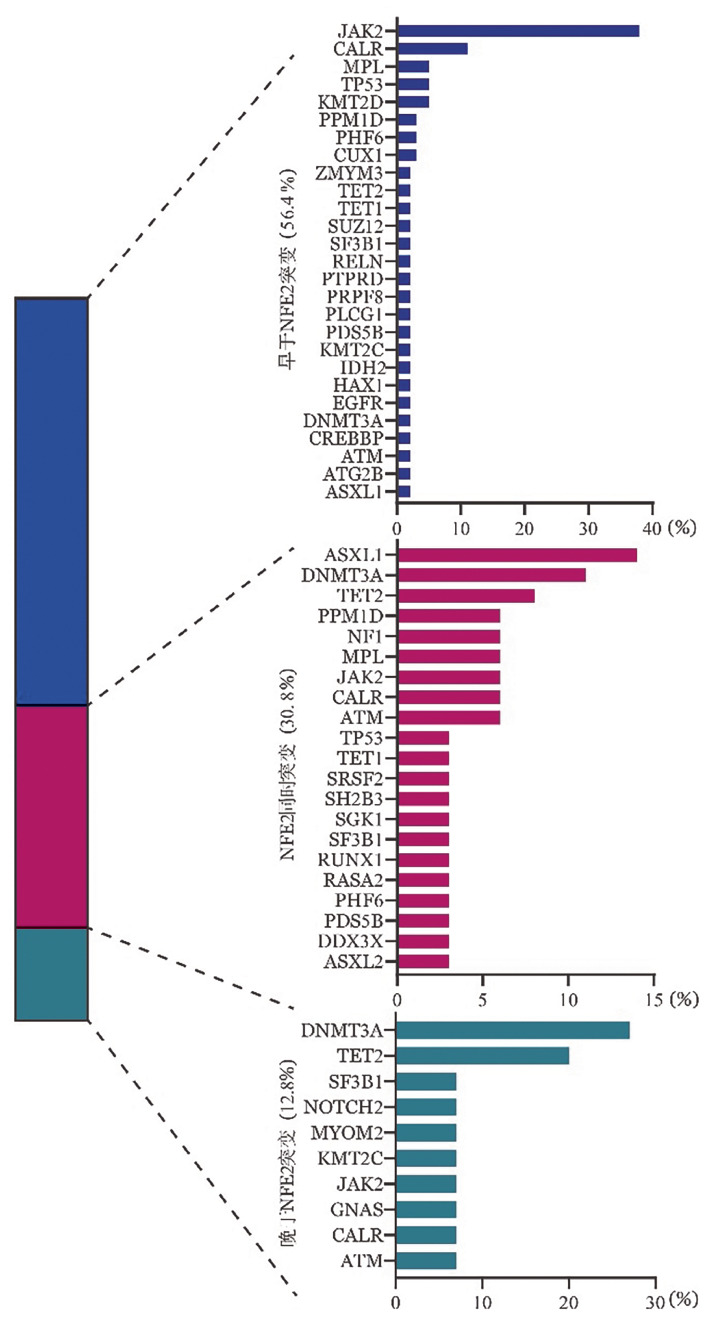
骨髓增殖性肿瘤患者NFE2基因突变共基因突变时序分析 **注** 利用等位基因突变频率（VAF）将共突变分类为早于、同时突变或晚于NFE2基因突变。左侧堆叠条表示各类别中共突变时序的分布，右侧条形图显示相应基因的百分比

3. 伴NFE2基因突变患者的临床和实验室特征：如表1所示，与无NFE2基因突变组比较，NFE2基因突变组年龄较大［中位年龄：60（54，67）岁对54（41，63）岁，*P*＝0.001］，诊断前血栓病史（39.0％对22.0％，*P*＝0.012）、诊断前动脉血栓病史患者比例（36.6％对20.4％，*P*＝0.014）均较高。NFE2基因突变组血清炎症因子IL-6（86.2％对65.0％，*P*＝0.019）、IL-10（75.9％对48.5％，*P*＝0.004）、TNF-α（75.9％对55.1％，*P*＝0.028）、IFN-γ（34.5％对17.5％，*P*＝0.022）增高的患者比例均高于无NFE2基因突变组。

**表1:** NFE2基因突变与无NFE2基因突变的骨髓增殖性肿瘤患者临床特征比较

临床特征	NFE2基因突变组（41例）	无NFE2基因突变组（682例）	统计量	*P*值
年龄［岁，*M*（*Q*_1_，*Q*_3_）］	60（54，67）	54（41，63）	*z*＝−3.239	0.001
女性［例（％）］	20（48.8）	379（55.6）	*χ*^2^＝0.721	0.396
诊断［例（％）］			–	0.873
PV	9（22.0）	150（22.0）		
ET	13（31.7）	247（36.2）		
PMF	14（34.1）	220（32.3）		
post-PV/ET MF	5（12.2）	65（9.5）		
WBC［×10^9^/L，*M*（*Q*_1_，*Q*_3_）］	7.7（4.9，10.9）	8.7（5.9，12.6）	*z*＝−1.413	0.158
NLR［*M*（*Q*_1_，*Q*_3_）］（695例）	4.2（2.9，8.0）	3.7（2.5，6.1）	*z*＝−1.495	0.135
HGB［g/L，*M*（*Q*_1_，*Q*_3_）］	135（104，160）	137（112，157）	*z*＝−0.100	0.920
HCT［％，*M*（*Q*_1_，*Q*_3_）］（712例）	41.5（32.2，47.6）	42.0（34.8，47.6）	*z*＝−0.082	0.935
PLT［×10^9^/L，*M*（*Q*_1_，*Q*_3_）］	582（239，775）	530（287，778）	*z*＝−0.017	0.987
EPO［U/L，*M*（*Q*_1_，*Q*_3_）］（691例）	8.3（2.2，31.0）	5.6（2.1，18.8）	*z*＝−0.896	0.370
LDH［U/L，*M*（*Q*_1_，*Q*_3_）］（693例）	339.5（238.8，441.3）	267.1（208.4，393.4）	*z*＝−1.844	0.065
体质性症状［例（％）］	3（7.3）	114（16.7）	*χ*^2^＝2.519	0.113
脾左肋缘下可触及［例（％）］（681例）	15（39.5）	259（40.3）	*χ*^2^＝0.010	0.922
循环中原始细胞≥1％［例（％）］（720例）	5（12.2）	86（12.7）	*χ*^2^＝0.008	0.930
诊断前血栓史［例（％）］	16（39.0）	150（22.0）	*χ*^2^＝6.341	0.012
诊断前动脉血栓病史［例（％）］	15（36.6）	139（20.4）	*χ*^2^＝6.058	0.014
诊断前静脉血栓病史［例（％）］	1（2.4）	17（2.5）	–	>0.050
骨髓纤维化分级［例（％）］			*χ*^2^＝0.457	0.499
MF-0/1级	25（61.0）	451（66.1）		
MF-2/3级	16（39.0）	231（33.9）		
细胞因子水平增高［例（％）］（503例）				
IL-1β	1（3.4）	30（6.3）	–	>0.050
IL-2	12（41.4）	133（28.1）	*χ*^2^＝2.363	0.124
IL-4	25（86.2）	351（74.1）	*χ*^2^＝2.140	0.144
IL-5	12（41.4）	192（40.5）	*χ*^2^＝0.009	0.926
IL-6	25（86.2）	308（65.0）	*χ*^2^＝5.504	0.019
IL-8	2（6.9）	39（8.2）	–	>0.050
IL-10	22（75.9）	230（48.5）	*χ*^2^＝8.170	0.004
IL-12P70	26（89.7）	348（73.4）	*χ*^2^＝3.778	0.052
IL-17	10（34.5）	93（19.6）	*χ*^2^＝3.707	0.054
TNF-α	22（75.9）	261（55.1）	*χ*^2^＝4.804	0.028
IFN-α	5（17.2）	57（12.0）	–	0.384
IFN-γ	10（34.5）	83（17.5）	*χ*^2^＝5.223	0.022
染色体核型［例（％）］（582例）				
异常核型	5（14.7）	70（12.8）	–	0.791
复杂核型	2（5.9）	9（1.6）	–	0.131
治疗方案［例（％）］（646例）				
羟基脲	14（42.4）	184（30.0）	*χ*^2^＝2.268	0.132
IFN	8（24.2）	203（33.1）	*χ*^2^＝1.121	0.290
芦可替尼	11（33.3）	136（22.2）	*χ*^2^＝2.214	0.137
抗血小板治疗	16（48.5）	285（46.5）	*χ*^2^＝0.050	0.823
抗凝治疗	1（3.0）	3（0.5）	–	0.190
移植治疗	0（0）	10（1.6）	–	>0.050

**注** PV：真性红细胞增多症；ET：原发性血小板增多症；PMF：原发性骨髓纤维化；post-PV/ET MF：真性红细胞增多症/原发性血小板增多症后骨髓纤维化；WBC：白细胞计数；NLR：中性粒细胞/淋巴细胞比值；HGB：血红蛋白；HCT：红细胞压积；PLT：血小板计数；EPO：促红细胞生成素；LDH：乳酸脱氢酶；IL：白细胞介素；TNF-α：肿瘤坏死因子-α；IFN：干扰素；–：使用Fisher确切概率法，无具体统计量

NFE2基因突变组患者有多项炎症细胞因子水平增高，将NFE2基因突变、年龄、合并疾病（包括慢性感染、肿瘤、自身免疫性疾病）纳入Logistic回归分析模型进行校正，结果表明NFE2基因突变是TNF-α（*OR*＝2.747，95％ *CI*：1.143～6.605，*P*＝0.024）、IFN-γ（*OR*＝2.689，95％ *CI*：1.191～6.076，*P*＝0.017）、IL-10（*OR*＝3.219，95％ *CI*：1.343～7.717，*P*＝0.009）、IL-12P70（*OR*＝3.397，95％ *CI*：1.003～11.508，*P*＝0.049）、IL-17（*OR*＝2.284，95％ *CI*：1.017～5.127，*P*＝0.045）增高的独立影响因素。

相较于NFE2非截短突变组（8例），NFE2截短突变组（31例）患者LDH水平更高（*P*＝0.014），脾左肋缘下可触及（*P*＝0.032）、骨髓纤维化分级≥2级（*P*＝0.034）的患者比例更高。2例患者同时有截短突变和非截短突变，因此未纳入分析，详见表2。

**表2:** NFE2截短突变与NFE2非截短突变的骨髓增殖性肿瘤患者临床特征比较

变量	NFE2截短突变（31例）	NFE2非截短突变（8例）	统计量	*P*值
年龄［岁，*M*（*Q*_1_, *Q*_3_）］	58（51，66）	66（60，73）	*z*＝−1.479	0.139
女性［例（％）］	12（38.7）	6（75.0）	–	0.112
诊断［例（％）］			–	0.228
PV	7（22.6）	2（25.0）		
ET	8（25.8）	5（62.5）		
PMF	11（35.5）	1（12.5）		
post-PV/ET MF	5（16.1）	0（0）		
WBC［×10^9^/L，*M*（*Q*_1_, *Q*_3_）］	7.8（4.3，11.0）	8.3（6.1，13.9）	*z*＝−0.522	0.602
NLR［*M*（*Q*_1_, *Q*_3_）］（35例）	4.4（2.8，8.5）	4.7（3.5，8.4）	*z*＝−0.162	0.871
HGB［g/L，*M*（*Q*_1_, *Q*_3_）］	137（98，160）	137（122，188）	*z*＝−0.626	0.531
HCT［％，*M*（*Q*_1_, *Q*_3_）］（36例）	42.2（32.5，48.8）	41.3（38.1，60.1）	*z*＝−0.495	0.621
PLT［×10^9^/L，*M*（*Q*_1_, *Q*_3_）］	572（186，820）	630（459，719）	*z*＝−0.209	0.835
EPO［U/L，*M*（*Q*_1_, *Q*_3_）］（37例）	8.2（2.0，35.0）	4.3（2.2，13.7）	*z*＝−0.737	0.461
LDH［U/L，*M*（*Q*_1_, *Q*_3_）］（38例）	363.3（252.1，527.2）	202.1（182.4，337.2）	*z*＝−2.466	0.014
体质性症状［例（％）］	3（9.7）	0（0）	–	>0.050
脾左肋缘下可触及［例（％）］（36例）	13（46.4）	0（0）	–	0.032
循环中原始细胞≥1％［例（％）］	5（16.1）	0（0）	–	0.563
诊断前血栓史［例（％）］	12（38.7）	4（50.0）	–	0.694
诊断前动脉血栓病史［例（％）］	11（35.5）	4（50.0）	–	0.686
诊断前静脉血栓病史［例（％）］	1（3.2）	0（0）	–	>0.050
骨髓纤维化分级［例（％）］			–	0.034
MF-0/1级	17（54.8）	8（100）		
MF-2/3级	14（45.2）	0（0）		
细胞因子水平增高［例（％）］（27例）				
IL-1β	1（4.3）	0（0）	–	>0.050
IL-2	9（39.1）	2（50.0）	–	>0.050
IL-4	20（87.0）	3（75.0）	–	0.495
IL-5	9（39.1）	3（75.0）	–	0.294
IL-6	19（82.6）	4（100）	–	>0.050
IL-8	1（4.3）	0（0）	–	>0.050
IL-10	18（78.3）	3（75.0）	–	>0.050
IL-12P70	21（91.3）	3（75.0）	–	0.395
IL-17	9（39.1）	0（0）	–	0.268
TNF-α	18（78.3）	3（75.0）	–	>0.050
IFN-α	4（17.4）	1（25.0）	–	>0.050
IFN-γ	8（34.8）	2（50.0）	–	0.613
染色体核型［例（％）］（32例）				
异常核型	4（16.7）	0（0）	–	0.550
复杂核型	2（8.3）	0（0）	–	>0.050

**注** PV：真性红细胞增多症；ET：原发性血小板增多症；PMF：原发性骨髓纤维化；post-PV/ET MF：真性红细胞增多症/原发性血小板增多症后骨髓纤维化；WBC：白细胞计数；NLR：中性粒细胞/淋巴细胞比值；HGB：血红蛋白；HCT：红细胞压积；PLT：血小板计数；EPO：促红细胞生成素；LDH：乳酸脱氢酶；IL：白细胞介素；TNF-α：肿瘤坏死因子-α；IFN：干扰素；–：使用Fisher确切概率法，无具体统计量

PV患者分别按照IWG-PV和MIPSS-PV进行预后分组，NFE2基因突变组高危患者比例高于无NFE2基因突变组（IWG-PV：66.7％对25.3％，*P*＝0.033；MIPSS-PV：22.2％对2.0％，*P*＝0.013）。按照IPSET和MIPSS-ET对ET患者进行分组，NFE2基因突变组和无NFE2基因突变组患者中IPSET高危患者比例分别为30.8％、15.4％（*P*＝0.259），MIPSS-ET高危患者比例依次是15.4％、6.1％（*P*＝0.021）。对PMF患者依次按照DIPSS、DIPSS-Chinese、DIPSS-plus、MIPSS-70、MIPSS70+version2、GIPSS判断预后，伴或不伴有NFE2基因突变的患者预后分组差异无统计学意义（均*P*>0.05）。此外，post-PV/ET MF患者MYSEC-PM预后评分组间差异无统计学意义（*P*＝0.194）。

4. NFE2基因突变对预后的影响：NFE2基因突变组中3例（7.9％，3/38）患者诊断后发生血栓，其中2例动脉血栓（脑梗死2例）、1例静脉血栓（肠系膜静脉血栓）；无NFE2基因突变组中42例（6.3％，42/671）患者诊断后发生血栓，组间累计血栓发生率差异无统计学意义（*P*＝0.555）。随访期间NFE2基因突变组患者未发生骨髓纤维化或白血病转化，因此本研究未能分析NFE2基因突变对于MPNs发生纤维化进展以及白血病转化的影响。

截至末次随访，NFE2基因突变组中位随访时间为31.6（15.7，72.2）个月，无NFE2基因突变组中位随访时间是26.8（18.3，36.3）个月（*P*＝0.112）。本研究中6.6％（47/709）患者死亡，其中NFE2基因突变组1例死亡、无NFE2基因突变组46例死亡。NFE2基因突变和无突变组5年OS率分别为97.1％（95％ *CI*：91.5％～100％）、88.1％（95％ *CI*：83.6％～92.9％），差异无统计学意义（*P*＝0.165）。在各诊断亚型中，PV、ET、PMF、post-PV/ET MF患者伴或不伴NFE2基因突变的OS差异均无统计学意义（均*P*>0.05）。NFE2截短突变组（31例）与无NFE2基因突变组（671例）OS差异无统计学意义（*P*＝0.282）。JAK2V617F和NFE2双基因突变（24例）与JAK2V617F基因突变不伴NFE2基因突变（459例）患者OS差异无统计学意义（*P*＝0.188）。

## 讨论

NFE2基因于20世纪80年代首次在红细胞中被发现，其在造血干细胞、髓系、红系和巨核系细胞中均有表达，对红系/巨核细胞发育和功能调节起重要作用[23]–[24]。研究表明，MPNs患者NFE2蛋白表达水平较正常人增高，部分MPNs患者伴NFE2基因杂合、截短突变，其野生型NFE2蛋白表达水平进一步增高[7],[9]–[10],[25]–[26]。造血系统特异性NFE2过表达和NFE2基因突变的转基因小鼠均表现出MPNs表型以及向急性髓系白血病和（或）髓系肉瘤转化的特征，研究者推测NFE2基因突变在MPNs发病和疾病进展中可能发挥作用[6],[27]。

国外研究报道5％左右MPNs患者存在NFE2基因突变，多数是移码突变，本研究结果与此相符[6]–[10]。既往研究提示PV患者NFE2基因突变频率略高于ET和PMF患者[7],[9]，但也有报道其在MF患者中频率更高[10]；本研究中MF患者NFE2基因突变频率略高于PV和ET，但差异无统计学意义，仍需大样本验证。NFE2基因突变位点分散，既往研究中NFE2基因突变多位于转录激活域和DNA结合域之间的区域（46％～75％）[7],[10]，本研究转录激活域突变频率最高（46.3％），其次是转录激活域和DNA结合域之间（24.4％）。尽管突变分布有一定差异，但MPNs中NFE2基因突变多为致C端截短的突变，影响DNA结合功能。NFE2基因突变最常位于第261号密码子（主要为E261Afs*3），Guglielmelli等[7]提出其为热点突变，本研究中该位点突变频率最高，第65号密码子突变频率次之。在Martín Castillo等[8]研究中3例伴有Y65*突变的骨髓增生异常综合征患者均表现为输血依赖、骨髓纤维化且有2例发生白血病转化。但局限于本研究NFE2基因突变病例数较少，尚未能进一步分析NFE2基因突变氨基酸位点对临床和预后的影响。MPNs中NFE2基因突变与其他基因突变的相关性研究较少。曾有报道MPNs患者中NFE2基因突变常与ASXL1、EZH2、TET2、KIT、SH2B3、CALR和MPL基因突变共同出现[7]。本研究证实MPNs患者中NFE2基因突变常与MPL基因突变共同出现，并发现NFE2基因突变还常与ATM、TET1、PPM1D基因突变共同出现。

此外，我们基于VAF分析发现多数NFE2基因突变为亚克隆突变，提示其在MPNs患者中为较晚发生的突变事件。驱动突变大多早于NFE2基因突变，与既往研究结果一致[8],[10]。但因VAF判断克隆演变存在局限性[12]，需单细胞或连续测序进一步验证。

NFE2基因突变对MPNs患者临床特征影响的研究较少。Guglielmelli等[7]研究发现NFE2基因突变的ET和PMF患者的确诊年龄较小，在Marcault等[9]研究中NFE2基因突变与MPNs确诊年龄无明显关联，而本研究中NFE2基因突变的患者年龄更大。曾有报道显示NFE2基因突变与红细胞压积水平升高和血小板计数减少相关[7],[9]，但本研究未证实。本研究结果显示，NFE2基因突变者诊断前血栓病史，主要是诊断前动脉血栓病史比例增高。NFE2基因突变的PV和ET患者预后分层上高危组比例增加。本研究与既往研究不一致的可能原因：①纳入人群年龄、人种的差异；②各个诊断亚型占比的差异；③基因突变位点不一致。以上结论还需增加病例后进行验证。Jutzi等[6]研究发现急性髓系白血病患者中不同于截短突变，部分NFE2错义突变不会增强NFE2野生型蛋白的功能，反而发挥显性负效应。本研究进一步分析NFE2截短突变患者相较于NFE2非截短突变患者的差异，结果显示NFE2截短突变患者LDH水平更高，脾左肋缘下可触及、骨髓纤维化分级≥2级的患者比例更高。

与无NFE2基因突变患者相比，携带NFE2基因突变患者表现出多种细胞因子水平增高，包括IL-12P70、IL-10、IL-17、TNF-α和IFN-γ。而炎症微环境在MPNs的发病和临床结局中具有重要作用。既往研究报道IL-12升高是PMF不良预后因素，IL-10在PMF中显著高于健康对照[28]。Cacemiro等[29]研究结果显示IL-17和IFN-γ在PMF中较ET中更高，而TNF-α可促进恶性克隆扩增[30]。因此，NFE2基因突变可能通过炎症信号和微环境重塑加速疾病进展。

既往研究报道在NFE2基因突变小鼠中观察到MPNs表型并向白血病转化的特征[6]，但在MPNs患者中NFE2基因突变对预后的意义仍未明确。Guglielmelli等[7]研究中伴有NFE2基因突变的ET患者进展为继发性纤维化的比例更高，伴有NFE2基因突变的PV、ET或PMF预后意义不明。Marcault等[9]研究中NFE2基因突变是MPNs患者转化为急性白血病和预后不良的危险因素。本研究因病例和随访限制，尚不能得出NFE2基因突变与疾病进展或生存的确切关系。已有报道[6]伴NFE2基因突变的PV患者可发生孤立性髓系肉瘤，且NFE2基因突变在孤立性髓系肉瘤患者中频率较高[31]，因此临床上对于NFE2基因突变的MPNs患者，不仅需要关注骨髓和外周血的原始细胞，还需警惕孤立性髓系肉瘤的发生。

综上，MPNs患者NFE2基因突变发生率约5％，多为移码突变和无义突变，在MPNs中是较晚发生的突变事件。NFE2基因突变的MPNs患者年龄较大，多个炎症因子水平升高（IL-10、IL-12P70、IL-17、TNF-α、IFN-γ）。本研究尚未得到NFE2基因突变对于MPNs患者进展纤维化、转化白血病和生存的意义，有待扩大病例和延长随访来进一步探索。此外，本研究是单中心、回顾性研究，所得结论需要多中心、前瞻性、长期随访研究验证。
